# Effect of Background Music on Attentional Control in Older and Young Adults

**DOI:** 10.3389/fpsyg.2020.557225

**Published:** 2020-10-20

**Authors:** Amélie Cloutier, Natalia B. Fernandez, Catherine Houde-Archambault, Nathalie Gosselin

**Affiliations:** ^1^International Laboratory for Brain, Music and Sound Research (BRAMS), Center for Research on Brain, Language and Music (CRBLM) and Laboratory for Music, Emotions and Cognition Research (MUSEC), Department of Psychology, University of Montreal, Montreal, QC, Canada; ^2^Laboratory of Behavioral Neurology and Imaging of Cognition (LabNIC) and Swiss Center for Affective Sciences (CISA), Department of Neuroscience, University of Geneva, Geneva, Switzerland; ^3^Laboratory of Cognitive and Affective Neuroscience (CANEURO), Department of Psychology, University of Zurich, Zurich, Switzerland

**Keywords:** healthy aging, executive functions, attentional control, flanker task, background music, musical emotions, arousal, neuropsychology

## Abstract

Healthy aging may be accompanied by cognitive decline that includes diminished attentional control, an executive function that allows us to focus our attention while inhibiting distractors. Previous studies have demonstrated that background music can enhance some executive functions in both young and older adults. According to the *Arousal-Mood Theory*, the beneficial influence of background music on cognitive performance would be related to its ability to increase the arousal level of the listeners and to improve their mood. Consequently, stimulating and pleasant music might enhance attentional control. Therefore, the aims of this study were (1) to determine if the influence of background music, and more specifically its arousal level, might improve attentional control in older adults and (2) whether this effect is similar across older and young adults. Older and young adults performed a visuo-spatial flanker task during three auditory conditions: stimulating music, relaxing music, and silence. Participants had to indicate as fast and as accurately as possible the direction of a central arrow, which was flanked by congruent or incongruent arrows. As expected, reaction times were slower for the incongruent compared to congruent trials. Interestingly, this difference was significantly greater under the relaxing music condition compared to other auditory conditions. This effect was the same across both age groups. In conclusion, relaxing music seems to interfere with visuo-spatial attentional control compared to stimulating music and silence, regardless of age.

## Introduction

Music listening induces strong and consistent emotions in the listener (Blood and Zatorre, 2001; Hunter and Schellenberg, 2010; Chanda and Levitin, 2013). As recommended by Eerola and Vuoskoski (2011), these musical emotions are often studied into valence (i.e., positive and negative emotions) and arousal (i.e., stimulating and relaxing) dimensions (Vieillard et al., 2008), which are taken from Russell’s model (Russell, 1980). Both music-resulting emotions and dimensions have been convincingly demonstrated to be associated with different musical parameters (for a review, see Juslin and Laukka, 2004). For example, most of the time, a fast tempo is associated with a high level of arousal, whereas a slow tempo is associated with a low level of arousal. Similarly, music composed in a major mode is typically associated with a high level of valence and with positive emotions like joy and peace. Also, it has been demonstrated that loudness is related to the perceived level of arousal and valence in music (Schubert, 2004; Dean et al., 2011; Olsen et al., 2015). Further studies also showed a positive correlation between the tempo of background music and reading speed (Kallinen, 2002), perceptual motor abilities (Nittono et al., 2000), and visual attention tasks (Bolger et al., 2013; Trost et al., 2014), giving an evident support to the impact of musical parameters on cognition. In Bolger et al. (2013) study, the targets of the visual attention task were presented across four selected metrical positions of the auditory stimulus in order to observe the entrainment effect of the rhythm of the music. In Trost et al. (2014) study, targets appeared time-locked to either strong or weak beats of the background music. The tempo of a musical stimulus presented before the cognitive task was also correlated to spatial ability (Husain et al., 2002).

Valence and arousal dimensions also seem to interact in inducing musical emotions (Salimpoor et al., 2009; van den Bosch et al., 2013). Previous work has demonstrated that music inducing a higher level of arousal generates more pleasure in the listener (Salimpoor et al., 2009). More precisely, if the listener likes the musical excerpt, subjective felt arousal ratings and the listener’s arousal state (as measured by electrodermal activity) increase with pleasure ratings (Salimpoor et al., 2009). These authors also specify that this link is unidirectional, since an increase in arousal does not always lead to pleasure (Salimpoor et al., 2009). Similarly, the familiarity of the music (i.e., how well someone knows the musical piece) has been positively correlated with the level of arousal and the pleasantness rated by the listener (van den Bosch et al., 2013). Moreover, it has been demonstrated that perception of positive emotional valence in music increases with age, with older adults tending to find music more pleasant on average than young adults (Cohrdes et al., 2020). It is also important to note that, in general, older adults have more positive emotional well-being than young adults (Carstensen et al., 2011).

Background music has been shown to have both beneficial and detrimental effects on a variety of cognitive functions in healthy young adults (see Kämpfe et al., 2010 for a meta-analysis). According to the most well-known theory regarding the link between music and cognitive performance, the *Arousal-Mood Theory*, a musical stimulus presented before the task and characterized by a high level of arousal (i.e., stimulating music) and a high level of valence (i.e., pleasant music) would increase the arousal level of the listener and improve his or her mood, thereby enhancing subsequent cognitive performance (Thompson et al., 2001). Other studies have demonstrated this effect when music was presented simultaneously with a variety of executive tasks, such as cognitive flexibility, working memory and attentional control (Thompson et al., 2005; Mammarella et al., 2007; Jefferies et al., 2008; Jiang et al., 2011; Bottiroli et al., 2014; Shih et al., 2016; Fernandez et al., 2020). However, not all of the available research findings fit well within this theoretical relationship between music and cognitive performance. For example, some research suggests that highly pleasant music requires more attentional resources and thus may impair cognitive performance in the context of attentional tasks (Nemati et al., 2019).

In particular, the impact of background music on attentional control, an executive function that allows one to focus attention on a specific stimulus, while inhibiting distractors from the environment (Theeuwes, 2010; Diamond, 2013), is somewhat ambiguous, with previous research generating heterogeneous results and not always controlling for levels of arousal and/or valence. One study demonstrated that, compared to silence, personally chosen background music enhanced young adults’ attentional control performance (Darrow et al., 2006), while another showed that sad music enhanced selective attention performance compared to calm, happy, and scary music (Jefferies et al., 2008). Furthermore, a study that used music prior to a flanker task, which measures attentional control (Eriksen and Eriksen, 1974), demonstrated that positive affect (i.e., higher valence level) induces a larger flanker effect [difference in reaction time (RT) between incongruent and congruent trials], suggesting that background music characterized by positive valence impairs attentional control performance (Rowe et al., 2007). A recent study reported improved perceptual judgment in a flanker task in young adults when they were listening to joyful and arousing background music, compared to sad and tender music as well as to silence (Fernandez et al., 2020); however, they did not find any effect of background music on attentional control performance *per se*. Also, Burkhard et al. (2018) showed that, compared to a silent condition, relaxing and exciting music did not have any effect on young adults’ inhibitory performance on the go/no-go task (Nosek and Banaji, 2001), nor on the event-related components underlying inhibitory processing. In sum, studies in young adults still show heterogeneous results concerning the effect of background music on attentional control, and the role of arousal in this effect is still not completely understood.

The role of music in attentional control in elderly populations has only been investigated very recently (Fernandez et al., 2020). In this work, they demonstrated that, compared to silence as well as sad and tender music, joyful and highly arousing background music enhanced perceptual judgments in a flanker task in both older and young adults (Fernandez et al., 2020). No background music effect was found on older adults’ attentional control performance (Fernandez et al., 2020). However, this study used a modified version of the flanker task, taken from the Attention Network Test, which measures several components of attention and includes cues before the trials. Thus, a more challenging task measuring attentional control specifically might produce different results regarding the effect of background music in older adults. In sum, it is apparent that the existing findings about the effect of background music on attentional control in both older and young adults are not always in accordance with the *Arousal-Mood Theory* (Thompson et al., 2001).

It is important to study the effect of background music in older adults since attentional control can be impaired in normal cognitive aging (Buckner, 2004). Indeed, older adults have been reported to be generally more sensitive to distractors (Hasher and Zacks, 1988; Hasher et al., 1991; Gazzaley et al., 2005; Darowski et al., 2008), showing slower RT in the flanker task compared to young adults (Zeef et al., 1996; Salthouse, 2010). Also, a speed-accuracy trade-off can be observed in older adults’ performance at the flanker task (Wild-Wall et al., 2008; Hsieh and Fang, 2012; Hsieh and Lin, 2014). In other words, compared to younger adults, older adults make fewer errors but present slower RT. It is important to note that this slowing in older adults’ performance could also be caused by vision and/or hearing loss, since a relationship has been demonstrated between vision and hearing and cognitive performance in this population (Li and Lindenberger, 2002; Wahl and Heyl, 2003; Lin et al., 2013). A recent study investigating the cerebral substrates associated with this age-related slowdown in the flanker task, demonstrated additional brain activations in posterior parieto-occipital areas which were linked to greater efforts to process the central target in incongruent trials (Fernandez et al., 2019). In sum, the literature indicates that older adults struggle more in inhibiting distractors and probably recruit different brain areas to compensate for their difficulties.

In addition to attentional declines in the elderly, it is possible that the presentation of a visual or auditory stimulus during the completion of a cognitive task might be more distracting for older adults than young adults, even if they are told to ignore the distraction (Guerreiro et al., 2010). Studies conducted by Alain and Woods (1999) and by Andrés et al. (2006) demonstrated that adding irrelevant sounds to a visual discrimination task impairs older adults more than young adults in their RT, as well as in the amplitude of the event-related potential linked to the processing of distraction (N1 and MMN). In the same manner, adding background music to a visual task could potentially be more distracting for older adults than for young adults.

However, it is also possible that background music added to a visual task could impair the performance of young adults. Indeed, for attentional tasks that are time-critical, as well as for spatial attentional tasks, shared attentional resources are involved when processing stimuli from different modalities (i.e., auditory and visual), and this is the case for adults of all ages (for a review, see Wahn and König, 2017). This can lead to impairment in the processing of one or both modalities. For example, when auditory and visual stimuli are presented simultaneously in an attentional task, both auditory and visual processing are slowed down (Dunifon et al., 2016). However, the effect of background music on attentional control in young and older adults is still not fully understood and needs further investigation.

In sum, normal aging is accompanied by cognitive decline that affects attentional control. Thus, it is important to find easy and pleasant ways for older adults to maximize their attentional control in everyday situations, for example with background music. However, the beneficial effect of background music on different executive functions is not fully understood, possibly due to the fact that the arousal level of music is not always controlled in previous studies. More particularly, the comparison between young and older adults in the effect of background music on attentional control specifically needs more investigation.

This study aimed to determine if the influence of background music, and more specifically its arousal level, might improve visuo-spatial attentional control in older adults and whether this effect is similar across older and young adults. To do this, we compared the effect of stimulating and relaxing music on performance on the flanker task, with a silence condition representing the base level performance.

Regarding the effect of background music, we expected faster answers and fewer errors for older adults under the stimulating music condition compared to both the relaxing music and silence conditions. As for young adults, knowing that results in the literature about the effect of background music on attentional control are still heterogeneous, there were no hypotheses concerning the effect of background music on their performance on the flanker task.

## Materials and Methods

### Participants

Nineteen older adults and 21 younger adults participated in this experiment. They all provided informed consent and received financial compensation for their participation. All participants were francophone Quebecers and reported to have normal audition, as well as normal or corrected-to-normal visual acuity. They also reported information about their music listening habits. None reported neurological, neurodevelopmental, or diagnosed psychiatric disorders. Depression and anxiety questionnaires were used to ensure that participants did not have clinically significant levels of anxio-depressive symptoms.

Young adults completed both the Beck Anxiety Inventory (BAI; Beck et al., 1988) and Beck Depression Inventory II (BDI-II; Beck et al., 1996), for which scores over critical thresholds (26/63 and 29/63, respectively) were considered exclusion criteria. All young adults presented scores of 12 or lower (*M* = 4.36; *SD* = 3.83) for the BAI and scores of 20 or lower (*M* = 7.24; *SD* = 5.32) for the BDI-II.

Older adults completed the Geriatric Anxiety Inventory (GAI; Pachana et al., 2007), as well as the short form of the Geriatric Depression Scale (GDS-SF; Burke et al., 1991). Scores over critical thresholds (9/20 and 5/15, respectively) were considered exclusion criteria. Older participants had scores of 8 or lower (*M* = 2.05, *SD* = 2.59) on the GAI, as well as of 3 or lower for the GDS-SF (*M* = 0.79; *SD* = 1.08). In addition, general cognitive state was evaluated using the Mini Mental State Examination (MMSE; Folstein et al., 1975; Commenges et al., 1992) to ensure no deficits (e.g., mild cognitive impairment). Based on previous studies (Folstein et al., 1975; Hudon et al., 2009), older participants whose scores were over the threshold of 27/30 were retained in the study. All older participants had scores of 28 or more (*M* = 29.26; *SD* = 0.73).

In addition, basic executive functioning was assessed using the color-word interference test, from the Delis-Kaplan Executive Function System battery (D-KEFS; Delis et al., 2001). The color-word interference condition consists of naming the color of the ink with which each word is printed, thus permitting an evaluation of inhibition processes. No inhibition deficits were observed in either group, with standard scores in the average range when compared to age-based norms (*M* = 10.29, *SD* = 1.82 for young adults; *M* = 10.42, *SD* = 1.98 for older adults).

The two groups were significantly different in age (see Table 1). They were matched in terms of sex, years of schooling, and years of musical training, with a similar proportion of men and women and equivalent years of schooling and years of musical training (see Table 1). However, our participants were mainly women (i.e., 18 older women for one man and 19 young women for two men). For musical expertise, none of the participants were professional musicians.

**Table 1:** Comparison between older and younger adults on demographic variables.

	Age groups					
	Older adults	Young adults	*df*	*t/χ*^2^	*p*	Effect size (*r*)
N (M, F)	19 (1, 18)	21 (2, 19)	1	0.26	=0.61	=0.08
Age (years)	67.26 (3.16)	23.95 (3.51)	38	−40.82	<0.001	=0.99
Years of education	16.16 (2.69)	16.48 (1.86)	38	0.44	=0.66	=0.07
Years of musical training	1.37 (2.17)	2.81 (4.69)	38	1.23	=0.23	=0.2

Except for sex, this table presents means (and standard deviations). M = male, F = female. Group composition was compared for sex, using a chi square test, and for age, years of education, and years of musical training using independant t-tests.

The music listening habits of our sample did not appear to be different between young and older adults, neither as principal activity (reported by 13/21 young adults with a mean of 2.69 h/week and 13/19 older adults with a mean of 2.76 h/week) nor as background music (reported by all young adults with a mean time of 8.1 h/week and 13/19 older adults with a mean time of 8.79 h/week).

### Flanker Task

**Figure 1:**
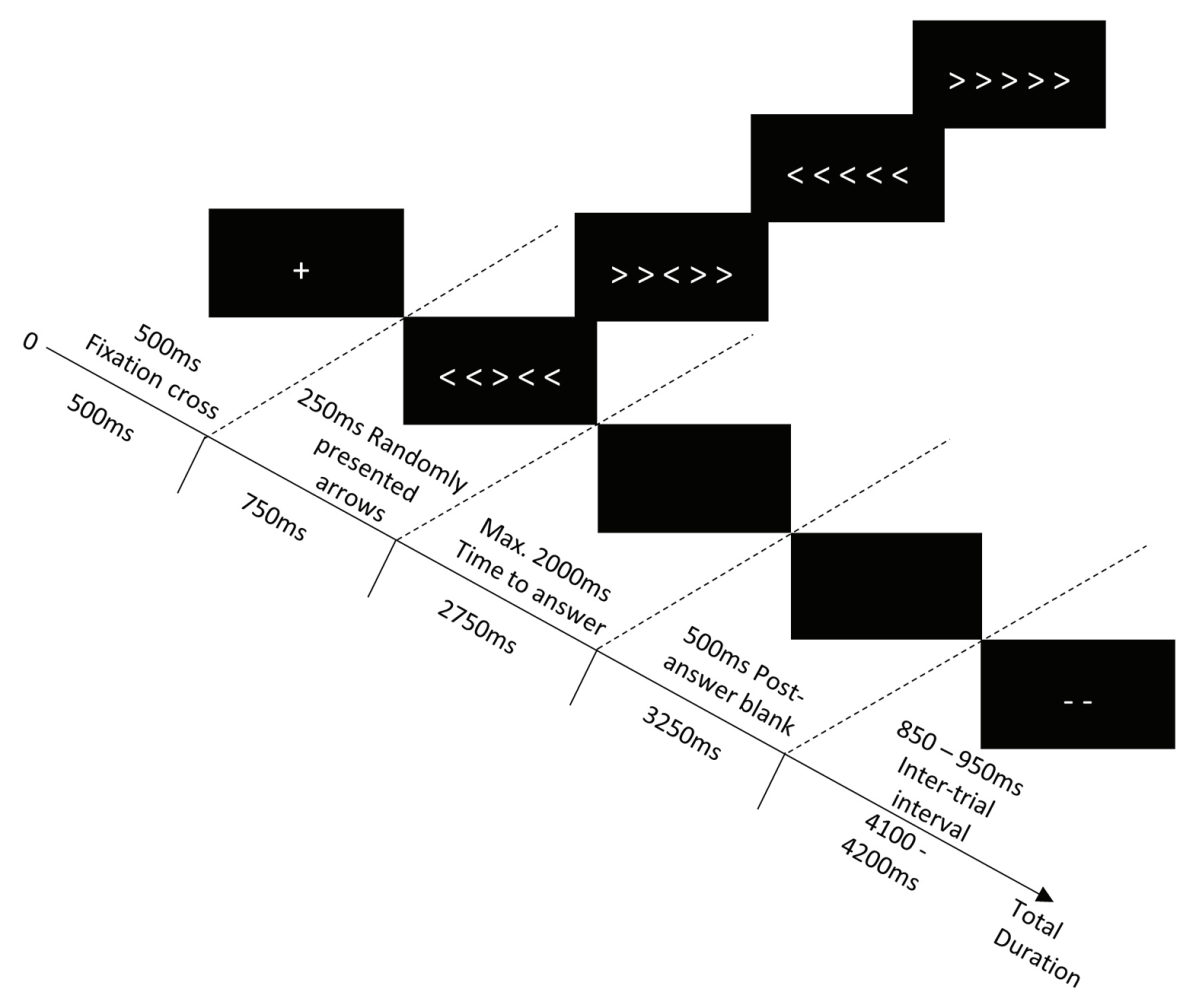
The course of a flanker task trial. Symbols are not to scale; they have been enlarged to be visible in this diagram.

All participants performed an arrow version of Eriksen’s flanker task (Eriksen and Eriksen, 1974) from a viewing distance of 100 cm from the screen. We followed previous recommendations for size and spacing parameters (Zeef et al., 1996; Maylor and Lavie, 1998; Hsieh and Lin, 2014). Participants were asked to focus their attention on the central arrow (0.4° of visual angle vertically and 0.6° horizontally) of a series of five and to indicate the direction in which it pointed, as quickly and accurately as possible. The target was flanked by two arrows on the left and two arrows on the right and could either point the same direction (congruent condition: right > > > > > or left < < < < <) or the opposite direction (incongruent condition: right < < > < < or left > > < > >) as the central arrow (see Figure 1).

Each trial contained five steps (see Figure 1). First, a fixation cross was displayed in the center of the screen for 500 ms, followed by an array of arrows in the middle of the screen for a duration of 250 ms. Next, a black screen was presented, and participants had a maximum of 2,000 ms to provide their answer regarding the direction of the central arrow. After the answer was given or the time limit was over, the screen remained black for 500 ms. Finally, the symbol “--” was presented in the middle of the screen during the inter-trial interval (duration between 850 and 950 ms). Depending on the participant’s RT and the duration of the inter-trial interval, the total duration of one trial varied between 2,500 and 4,200 ms.

The experiment comprised 21 blocks containing 32 trials each (with an equal number of congruent and incongruent trials) for a total of 672 trials. Both blocks and trials were presented in a randomized order for each participant. Of the 21 blocks, seven were allocated to each of the three auditory conditions (stimulating music, relaxing music, and silence). For the two musical conditions, each block was associated with a different musical excerpt. Participants could take breaks between each block to rest. The total duration of one block varied between 80 and 90 s, depending on the RT of the participant. Without the breaks between each block, the total duration of the entire task was approximately 30 min. To familiarize participants with the task, it was preceded by a practice block that included feedback to inform the participants about their performance. The practice block was presented with background music characterized by an intermediate tempo (i.e., 110 beats per minute, BPM). The flanker task and the music were presented using MATLAB (MATLAB Release 2018a, The MathWorks, Inc., Natick, Massachussetts, United States) with the “Psychophysics Toolbox Version 3” extension (Brainard, 1997; Kleiner et al., 2007).

### Musical Stimuli

All participants performed the flanker task under three auditory conditions: stimulating music, relaxing music, and silence. The music was pleasant sounding instrumental works composed in a major mode, chosen from the classical repertoire. Inter-rater agreement between three researchers was used to select the seven most stimulating (e.g., William Tell Overture: Final, composed by Giochino Rossini), as well as the seven most relaxing (e.g., Suite Bergamasque, Clair de Lune composed by Claude Debussy), musical excerpts from a larger pool of musical material in use in our laboratory. Excerpts of 100 s were chosen from the original pieces, so that the arousal and valence levels, as well as the tempi, were stable throughout each excerpt. The stimulating musical excerpts had a mean tempo of 153.14 BPM (*SD* = 23.35), while the relaxing musical excerpts had a mean tempo of 59.29 BPM (*SD* = 11.34). All excerpts were normalized at peak value (90% of maximum amplitude) and logarithmic fade-ins and fade-outs of 500 ms were added at the beginning and end of each excerpt, using Adobe Audition 3.0 software (Adobe Systems, Inc. San Jose, CA, United States). The music was presented *via* Beyer Dynamic Headphones (Model DT 770 Professional, 250 OHM).

### Musical Evaluation

After completing the flanker task, participants were asked to listen carefully to each musical excerpt without time restriction and to evaluate how much the piece was considered to be (a) arousing, i.e., relaxing or stimulating, (b) unpleasant or pleasant, and (c) unfamiliar or familiar, using a continuous visual analogue scale from 0 (extreme left) to 100 (extreme right). Thus, a low score on the arousal dimension would mean that the musical excerpt was judged as relaxing.

### Data Analysis

Group composition was compared for sex, using a chi square test, and for age, years of education, and years of musical training using independent *t*-tests.

Performance on the flanker task was analyzed using RT and error rate (ER). Average RT values for successful trials were calculated in milliseconds for each flanker congruency type of trial (i.e., congruent and incongruent), each auditory condition (stimulating music, relaxing music, and silence), and each participant separately. The averages and standard deviations of ER as percentages (excluding missed trials) were also calculated for each participant, as well as for each flanker congruency and auditory condition. RT and ER scores were entered into separate mixed-design analyses of variance (ANOVAs) with Age Group (older and young adults) as a between-subject factor, Auditory Condition (stimulating music, relaxing music, and silence), and Flanker Congruency trial type (congruent and incongruent) as within-subject factors. When interactions between repeated measure factors were significant, a standard contrasts analysis was used to determine if the difference between congruent and incongruent trials (i.e., flanker effect) was the same between auditory conditions.

To confirm that the musical conditions differed in perceived arousal level and to explore whether there was a difference between older and younger adults’ judgments, a mixed-design ANOVA with the between subject factor Age Group (older and younger adults) and the within subject factor Music Condition (stimulating music and relaxing music) was conducted. Two other exploratory mixed design ANOVAs were conducted with the judgments of valence and familiarity.

All statistical analyses were performed using IBM SPSS Statistics 24 (IBM Corp., 2016). Behavioral mean results (music evaluation and flanker performance) as well as statistical results are presented in Tables 1–4.

**Table 2:** Results of the analyses of variance (ANOVA) for the evaluation of arousal, valence, and familiarity.

	Predictor	*df*	*F*	*p*	*η*^2^
	**Arousal**Music Condition Age Group Music Condition × Age Group	1, 38 1, 38 1, 38	1453.3 0.016 2	<0.001 =0.9 =0.165	0.98 0.00 0.05
	ValenceMusic Condition Age Group Music Condition × Age Group	1, 38 1, 38 1, 38	32.28 7.53 0.009	<0.001 =0.009 =0.926	0.46 0.17 0.00
	FamiliarityMusic Condition Age Group Music Condition × Age Group	1, 38 1, 38 1, 38	1.32 21.48 0.08	=0.258 <0.001 0.778	0.033 0.36 0.002

**Table 3:** Results of the ANOVA for the flanker task RT.

	Predictor	*df*	*F*	*p*	*η*^2^
	Omnibus analysisAge Group Flanker Congruency Auditory Condition × Flanker Congruency	1, 38 1, 38 2, 67	55.02 418.75 3.995	<0.001 <0.001 0.027	0.59 0.92 0.095
	Contrasts analysisRelaxing vs. Stimulating Relaxing vs. Silence Stimulating vs. Silence	1, 38 1, 38 1, 38	10.61 4.29 0.116	=0.002 =0.045 0.735	0.22 0.1 0.003

**Table 4:** Results of the ANOVA for the flanker task ER.

	Predictor	*df*	*F*	*p*	*η*^2^
	Omnibus analysisAge Group Flanker Congruency Age Group × Flanker Congruency Auditory Condition	1, 38 1, 38 1, 38 2, 38	9.86 66.28 14.05 2.46	=0.003 <0.001 <0.001 =0.097	0.21 0.64 0.27 0.056
	*Post-hoc* analysis (ANOVAs)Difference between older and young adults for congruent trials Difference between older and young adults for incongruent trials	1, 38 1, 38	1.13 11.45	=0.294 =0.002	0.03 0.23

## Results

### Musical Stimuli Evaluation

**Figure 2:**
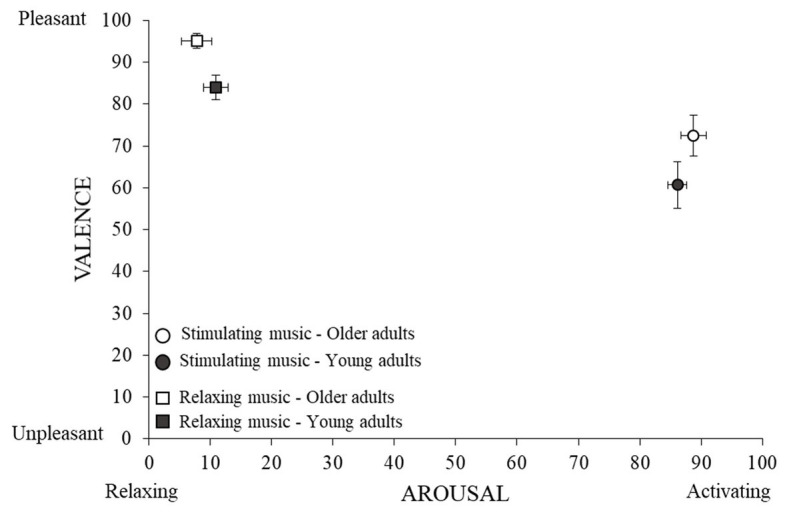
Judgments of arousal and valence. Mean rating (and standard errors) are presented as a function of music conditions and age groups on both valence and arousal dimensions.

As expected, stimulating music was judged to be significantly more arousing than relaxing music by both older and young adult groups, the size of this effect being large (see Figure 2 and Table 2). There was no difference between older and young adults in their evaluation of the arousal level of musical excerpts and no significant interaction between Music Condition and Age Group.

Relaxing music was considered significantly more pleasant than stimulating music by both older and young adults, with this effect being large (see Figure 2 and Table 2). Older adults generally judged the musical excerpts to be more pleasant than young adults, with this effect also being large. There was no significant interaction between Music Condition and the Age Group.

Older adults were significantly more familiar (*M* = 84.41, *SD* = 16.73) with the musical excerpts than young adults (*M* = 62.32, *SD* = 17.41), with the size of this effect being large (see Table 2). There was no difference between stimulating and relaxing music in their level of familiarity. Finally, there was no significant interaction between Music Condition and the Age Group.

### Flanker Task

**Figure 3:**
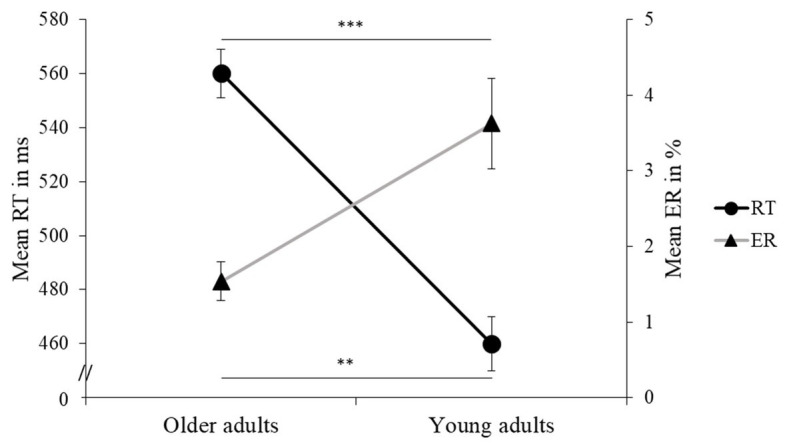
Mean reaction time (RT) in ms and error rate (ER) in % (and standard errors) are presented for older and young adults. Values of *p* (asterisk): ^**^p < 0.01 and ^***^p < 0.001.

**Figure 4:**
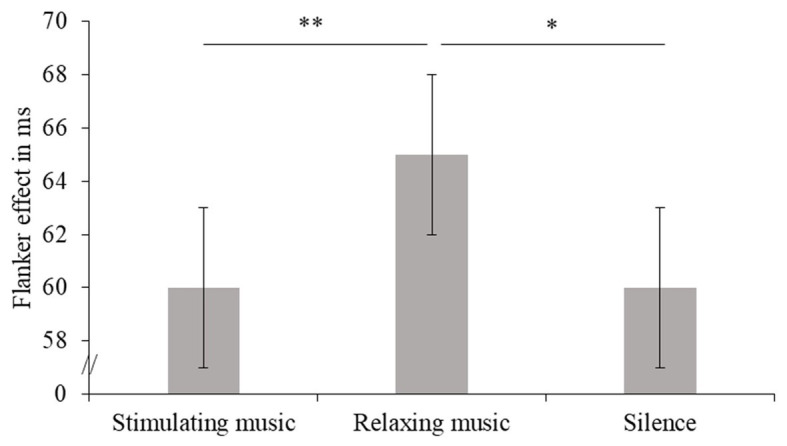
Flanker effects in ms (and standard errors) are presented for all participants (combined across age groups) as a function of Auditory Condition. Values of *p* (asterisk): ^*^p < 0.05 and ^**^p < 0.01.

Reaction time performance on the flanker task revealed a significant and general slowing in older adults compared to young adults (large effect, see Table 3 and Figure 3). For both older and young adults, RT was significantly slower in the incongruent trials than in the congruent ones, i.e., a flanker effect was clearly observed. Moreover, an interaction between Auditory Condition and Flanker Congruency showed that the difference in RT between incongruent and congruent trials varied between the three conditions. More specifically, the influence of background music revealed a greater flanker effect for relaxing music than for stimulating music or silence, these effects being, respectively, large and average (see Figure 4 and Table 3). These two latter conditions did not differ in terms of flanker effect (see Figure 4 and Table 3).

**Figure 5:**
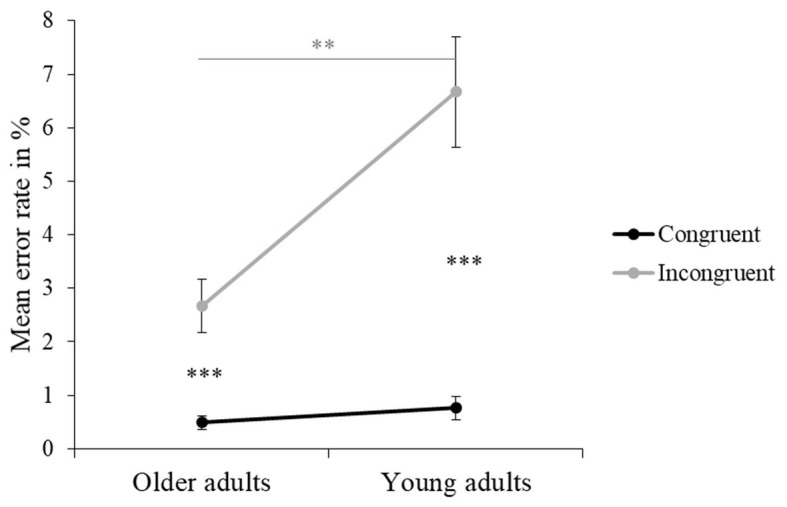
Interaction between Age Group and Flanker Congruency in ER. Mean ER in % (and standard errors) were obtained for congruent and incongruent trials and separately for older and young adults. Values of *p* (asterisk): ^**^p <0.01 and ^***^p < 0.001.

Older adults made fewer errors overall compared to young adults, and this was a large effect (see Figure 3 and Table 4). Also, for both older and young adults, ER was significantly higher for the incongruent than the congruent trials, this effect also being large. Moreover, an interaction between Age Group and Flanker Congruency showed that there was no age-related difference for the congruent trials, while older adults made significantly fewer errors in the incongruent trials compared to young adults (see Figure 5 and Table 4). There were no significant differences between the three experimental conditions in ER.

## Discussion

This study aimed to explore the effect of the arousal level of background music on visuo-spatial attentional control in young and older adults. To do this, both groups performed an arrow version of the flanker task under three auditory conditions: stimulating music, relaxing music, and silence.

### Error Rates and Reaction Times in the Flanker Task

The effects on ER seemed quite limited, probably due to the ceiling effect observed in both older and young adults (success rate > 95% for all participants). All participants presented expected slower RT and increased errors in the incongruent trials compared to the congruent ones, suggesting greater difficulty in inhibiting the distracting and incongruent arrows (Eriksen and Eriksen, 1974; Eriksen, 1995). Importantly, older adults had overall slower RT and lower ER than young adults. They seemed to favor accuracy over speed in their performance on the flanker task, while young adults favored speed over accuracy, which is consistent with previous studies using the same task (Wild-Wall et al., 2008; Hsieh and Fang, 2012; Hsieh and Lin, 2014). Although normal aging has been linked to impairments in attentional control (Hasher and Zacks, 1988; Hasher et al., 1991; Buckner, 2004; Gazzaley et al., 2005; Darowski et al., 2008), the slower results observed in our older adults during incongruent trials might be explained by compensatory mechanisms adopted to adequately complete the task (Wild-Wall et al., 2008; Hsieh and Fang, 2012; Hsieh and Lin, 2014), or decreased eyesight and hearing, which has been linked to cognitive performance deficits (Li and Lindenberger, 2002; Wahl and Heyl, 2003; Lin et al., 2013).

In addition, results obtained in the flanker task might have been influenced by the female dominance of our sample. A previous study demonstrated that visual selective attention performance of women is more affected by invalid cues, while men benefit from those invalid cues (Merritt et al., 2007). Also, women are more influenced by irrelevant spatial cues compared to men (Bayliss et al., 2005). Finally, a study demonstrated that incongruent flankers impair women’s performance more than men’s, showing a gender difference in visuo-spatial selective attention (Stoet, 2010).

### The Effect of Background Music on Attentional Control

In our study, the influence of background music during a visuo-spatial attention task revealed impaired attentional control performance during relaxing music exposure compared to silence and stimulating music. These results are not consistent with the *Arousal-Mood Theory* (i.e., stimuli rated as pleasant and stimulating can increase the arousal level and improve the mood in listeners) and other previous studies demonstrating that stimulating and pleasant music enhances cognitive performance (Thompson et al., 2001; Mammarella et al., 2007; Jiang et al., 2011; Bottiroli et al., 2014; Shih et al., 2016; Fernandez et al., 2020). They are also inconsistent with recent research that suggests no influence of relaxing or stimulating background music on inhibitory processing, albeit using a different type of inhibition task (go/no-go; Burkhard et al., 2018).

It is difficult to reconcile these results demonstrating a difference between relaxing and stimulating music with the existing literature. However, based on previous studies demonstrating positive correlations between the tempo of music and cognitive performance across a number of domains, including reading speed (Kallinen, 2002), perceptual motor speed (Nittono et al., 2000), spatial ability (Husain et al., 2002), and visual attention tasks (Bolger et al., 2013; Trost et al., 2014), we hypothesize that this influence of relaxing music on participants’ reaction times might be associated with the tempi of our musical excerpts. Greater flanker effects are observed for the relaxing music condition, which is characterized by slower tempi, compared to the stimulating music condition, associated with faster tempi.

The contradiction between our results and the *Arousal-Mood Theory* may also be explained by the fact that, in this study, stimulating music was judged to be less pleasant than relaxing music, while usually it is judged to be more pleasant (Salimpoor et al., 2009). In line with this, a previous study that used music to induced different moods prior the flanker task, showed that pleasant music induced a general slowdown in RT, as well as a greater flanker effect, compared to both neutral condition and unpleasant music (Rowe et al., 2007). Although all of the musical excerpts in our study were judged to be pleasant, the fact that relaxing music was seen as significantly more pleasant than stimulating music might explain why the former induced a greater flanker effect than the latter. It has also been demonstrated that listening to highly pleasurable music involves more attentional resources, and leads to a decline in cognitive performance (Nemati et al., 2019). Thus, it is possible that our relaxing music impaired the flanker task performance compared to stimulating music and silence by inducing a higher positive valence in our participants and by the same token, involving more attentional resources, leaving less for the execution of the task (Dunifon et al., 2016; Wahn and König, 2017; Nemati et al., 2019). It is possible that the sharing of attentional resources when processing both auditory and visual stimuli at the same time is even more difficult when the auditory stimuli is relaxing music (Dunifon et al., 2016; Wahn and König, 2017; Nemati et al., 2019).

### The Effect of Background Music Across Age

Regarding age, the greater flanker effects observed for the relaxing music condition was similar between both groups. This is in line with very recent work demonstrating that classical music with different arousal and valence levels has the same impact on attention processing in both young and older adults, even if the latter experience a decline in this particular executive function (Fernandez et al., 2020). This also mirrors a study by Alain and Woods (1999), who reported no differences between older and young adults in their performance on a visual attention task, while listening to irrelevant auditory stimuli. However, in their study, the irrelevant sounds presented simultaneously with the visual attention task provoked greater event-related potential amplitude in older adults compared to young adults. These results demonstrate distinct background sounds processing across age for similar performances in a visual attention task. The behavioral similarities between older and young adults in the current study cannot rule out the possibility of age-related differences in the underlying neural networks for attentional control. Moreover, given that older and young adults differed in rating the musical excerpts for valence and familiarity, it is possible that, with similar ratings in these two dimensions, we would observe a difference between the two age groups in the effect of background music on attentional control.

### Musical Stimuli Evaluation

We analyzed participants’ arousal, valence, and familiarity evaluations of the musical excerpts. Our results indicated that the arousal level of the musical excerpts was judged as expected by both age groups. Also, all musical excerpts were evaluated as pleasant, but unexpectedly, relaxing music pieces were felt to be more pleasant than stimulating music pieces. This finding is inconsistent with previous studies demonstrating that stimulating music generated higher ratings of pleasantness by listeners (Salimpoor et al., 2009; van den Bosch et al., 2013). However, those studies used musical material chosen by the participants or that resembled participants’ favorite music, from various musical genres (classic, jazz, rock, etc.), whereas in our study, only classical music selected by the researchers was used. Indeed, it has been demonstrated that, when listening to familiar music, there is a strong positive correlation between the pleasure felt by the listener and his level of arousal, but when the music is not familiar, there is no longer a clear relation between pleasure and arousal (van den Bosch et al., 2013). This might explain why we obtained different results, since our participants listened to music that they did not choose and were thus not as familiar as they would have been with personally chosen music.

Relaxing and stimulating music did not differ in terms of familiarity level here, suggesting that the observed effect of background music on attentional control is likely due to the variations in arousal and valence levels only. However, we did find that music excerpts used in the current experiment were rated as more familiar and more pleasant for older adults than young adults. These results might be explained by the fact that older adults listen more to classical music, while young adults listen mostly to popular music (Savage, 2006). Thus, our musical excerpts might have matched older adults’ tastes and habits better than young adults’. Another putative explanation is related to the fact that perception of positive emotional valence in music increases with age; in other words, older adults tend to find music more pleasant on average than young adults (Cohrdes et al., 2020). The authors of this study interpret this finding in relation to other research that suggests that older adults’ emotional well-being is more positive than young adults’ (Carstensen et al., 2011). Hence, it is possible that our older participants were more inclined to find the music pleasant than our younger participants due to their age.

Moreover, our participants were mostly women, and gender is known to have a moderate influence on the emotions induced by music (Aljanaki et al., 2016). Indeed, women tend to feel more amazed by classical music than men (Aljanaki et al., 2016). It has been showed that the brain activity linked to music-induced pleasantness and self-reported feelings of happiness were significantly greater in women than in men (Diaz et al., 2011). Another study demonstrated that women show elevated electrophysiological activity for arousing and unpleasant music, compared to men (Nater et al., 2006). Thus, the female dominance in our study might have influenced the results concerning musical stimuli evaluation.

Although the evaluation of the valence dimension showed unexpected results [i.e., (1) relaxing music evaluated as more pleasant than stimulating music and (2) overall higher valence scores in the older compared to younger adults], the evaluation of the arousal dimension of our stimulating/relaxing musical excerpts were judged as expected. This allowed us to evaluate the effect of the arousal dimension of background music on attentional control, as measured by the flanker task.

### Conclusion

In conclusion, we observed the expected performance of older and young adults in the flanker task, with slower RT and greater ER for incongruent trials compared to congruent trials. Our results, not supported by the *Arousal-Mood Theory*, suggest that relaxing pleasant background music can impair visuo-spatial attentional control performance, inducing a greater flanker effect or RT than that observed for stimulating pleasant music and silence. This effect was the same for older and young adults despite the typical decrement in attentional control associated with healthy aging.

### Limitations

This study presents some limitations. First, only classical music was used and older adults found it more pleasant and familiar than young adults, potentially inducing a differential impact of these stimuli on our participants as a function of age. Future studies should control for the impact of age on emotional judgments of musical stimuli when comparing older and young adults. Second, although we screened older adults for cognitive impairments, given that hearing and vision loss can impact the cognitive performance of older adults, ideally these perceptual functions should be measured as well and, if necessary, entered as covariates in the analysis. Third, the music conditions differed not only in arousal but also in valence. Even if those two dimensions often interact together (Jefferies et al., 2008), it would be interesting to observe their separate effects on attentional control performance through manipulation of each of these factors independently. Fourth, given that a single visuo-spatial task was used to assess attentional control, the conclusions of this study are limited to visuo-spatial attentional control.

### Future Perspectives

In order to improve the evaluation of arousal, future studies should use real-time objective measurements of arousal through the recording of electrodermal activity, while participants listen to the music and execute the task. Also, it would be interesting to control for inter-individual variability, in general, arousal level by measuring it before the beginning of the experiment. It would also be important to take into account the gender of participants in studying music-induced emotions. Moreover, to improve the ecological validity of the results, future work could also investigate the influence of longer periods of background music listening (in contrast to our 100 s excerpts) on visuo-spatial attentional control performance. An important extension to the current research would be the inclusion of other modalities of attentional control during background music listening, in order to draw more general conclusions about attentional control, and not limited to visuo-spatial attentional control as in the current paper. As mentioned previously, since some studies found an effect of background music on cortical activity in absence of a behavioral effect (Alain and Woods, 1999; Jäncke and Sandmann, 2010), it would also be interesting to investigate the impact of background music on EEG measures. Finally, if future studies support the present findings demonstrating a detrimental effect of relaxing background music on visuo-spatial attentional control performance and reproduce this effect with other executive functions, this study should be used as guideline in recommending or not the use of background music, while performing a cognitive task.

## Data Availability Statement

The raw data supporting the conclusions of this article will be made available by the authors, without undue reservation.

## Ethics Statement

The studies involving human participants were reviewed and approved by Comité d’éthique de la recherche en arts et en sciences, Université de Montréal. The participants provided their written informed consent to participate in this study.

## Author Contributions

AC elaborated the theoretical frame and formulated the research question, as well as the objectives and hypotheses. AC contributed to the creation of the research protocol and methodology. AC contributed to the data collection and analysis and wrote the article. CH-A contributed to the creation of the research protocol and methodology, as well as the data collection. CH-A contributed to the revision and correction of the article. NF contributed to the establishment of the flanker task parameters, as well as to the revision and correction of the article. NG contributed to the elaboration of the theoretical frame and the formulation of the research question, objectives, and hypotheses. NG supervised the creation of the research protocol and methodology, as well as the data collection and analysis and the article redaction. All authors contributed to the article and approved the submitted version.

### Conflict of Interest

The authors declare that the research was conducted in the absence of any commercial or financial relationships that could be construed as a potential conflict of interest.

## References

[ref1] Alain C. Woods D. L. (1999). Age-related changes in processing auditory stimuli during visual attention: evidence for deficits in inhibitory control and sensory memory. Psychol. Aging 14, 507–519. 10.1037/0882-7974.14.3.507, PMID: 10509703

[ref72] Aljanaki A. Wiering F. Veltkamp R. C. (2016). Studying emotion induced by music through a crowdsourcing game. Inf. Process. Manag. 52, 115–128. 10.1016/j.ipm.2015.03.004

[ref2] Andrés P. Parmentier F. B. Escera C. (2006). The effect of age on involuntary capture of attention by irrelevant sounds: a test of the frontal hypothesis of aging. Neuropsychologia 44, 2564–2568. 10.1016/j.neuropsychologia.2006.05.005, PMID: 16797613

[ref3] Bayliss A. P. di Pellegrino G. Tipper S. P. (2005). Sex differences in eye gaze and symbolic cueing of attention. Q. J. Exp. Psychol. A 58, 631–650. 10.1080/02724980443000124, PMID: 16104099

[ref4] Beck A. T. Epstein N. Brown G. Steer R. A. (1988). An inventory for measuring clinical anxiety: psychometric properties. J. Consult. Clin. Psychol. 56, 893–897. 10.1037//0022-006x.56.6.893, PMID: 3204199

[ref5] Beck A. T. Steer R. A. Brown G. K. (1996). Manual for the beck depression inventory-II. San Antonio, TX: Psychological Corporation.

[ref6] Blood A. J. Zatorre R. J. (2001). Intensely pleasurable responses to music correlate with activity in brain regions implicated in reward and emotion. Proc. Natl. Acad. Sci. U. S. A. 98, 11818–11823. 10.1073/pnas.191355898, PMID: 11573015 PMC58814

[ref7] Bolger D. Trost W. Schön D. (2013). Rhythm implicitly affects temporal orienting of attention across modalities. Acta Psychol. 142, 238–244. 10.1016/j.actpsy.2012.11.012, PMID: 23357092

[ref8] Bottiroli S. Rosi A. Russo R. Vecchi T. Cavallini E. (2014). The cognitive effects of listening to background music on older adults: processing speed improves with upbeat music, while memory seems to benefit from both upbeat and downbeat music. Front. Aging Neurosci. 6:284. 10.3389/fnagi.2014.00284, PMID: 25360112 PMC4197792

[ref73] Brainard D. H. (1997). The psychophysics toolbox. Spat. Vis. 10, 433–436. 10.1163/156856897X00357 9176952

[ref9] Buckner R. L. (2004). Memory and executive function in aging and AD: multiple factors that cause decline and reserve factors that compensate. Neuron 44, 195–208. 10.1016/j.neuron.2004.09.006, PMID: 15450170

[ref10] Burke W. J. Roccaforte W. H. Wengel S. P. (1991). The short form of the Geriatric Depression Scale: a comparison with the 30-item form. J. Geriatr. Psychiatry Neurol. 4, 173–178. 10.1177/089198879100400310, PMID: 1953971

[ref11] Burkhard A. Elmer S. Kara D. Brauchli C. Jäncke L. (2018). The effect of background music on inhibitory functions: an ERP study. Front. Hum. Neurosci. 12:293. 10.3389/fnhum.2018.00293, PMID: 30083099 PMC6064730

[ref12] Carstensen L. L. Turan B. Scheibe S. Ram N. Ersner-Hershfield H. Samanez-Larkin G. R.. (2011). Emotional experience improves with age: evidence based on over 10 years of experience sampling. Psychol. Aging 26, 21–33. 10.1037/a0021285, PMID: 20973600 PMC3332527

[ref13] Chanda M. L. Levitin D. J. (2013). The neurochemistry of music. Trends Cogn. Sci. 17, 179–193. 10.1016/j.tics.2013.02.007, PMID: 23541122

[ref15] Cohrdes C. Wrzus C. Wald-Fuhrmann M. Riediger M. (2020). “The sound of affect”: age differences in perceiving valence and arousal in music and their relation to music characteristics and momentary mood. Music. Sci. 24, 21–43. 10.1177/1029864918765613

[ref16] Commenges D. Gagnon M. Letenneur L. Dartigues J. -F. Barberger-Gateau P. Salamon R. (1992). Statistical description of the Mini-Mental State Examination for French elderly community residents. J. Nerv. Ment. Dis. 180, 28–32. 10.1097/00005053-199201000-00007, PMID: 1538203

[ref17] Darowski E. S. Helder E. Zacks R. T. Hasher L. Hambrick D. Z. (2008). Age-related differences in cognition: the role of distraction control. Neuropsychology 22, 638–644. 10.1037/0894-4105.22.5.638, PMID: 18763883

[ref18] Darrow A.-A. Johnson C. Agnew S. Fuller E. R. Uchisaka M. (2006). Effect of preferred music as a distraction on music majors’ and nonmusic majors’ selective attention. Bull. Counc. Res. Music. Educ. 170, 21–31.

[ref19] Dean R. T. Bailes F. Schubert E. (2011). Acoustic intensity causes perceived changes in arousal levels in music: an experimental investigation. PLoS One 6:e18591. 10.1371/journal.pone.0018591, PMID: 21533095 PMC3080387

[ref20] Delis D. C. Kaplan E. Kramer J. H. (2001). Delis-Kaplan executive function system. 10.1017/S1355617704102191 15012851

[ref21] Diamond A. (2013). Executive functions. Annu. Rev. Psychol. 64, 135–168. 10.1146/annurev-psych-113011-143750 23020641 PMC4084861

[ref22] Diaz J.-L. Flores-Gutiérrez E. O. Rio-Portilla Y. Cabrera M. C. (2011). “Musical emotion assessment, brain correlates, and gender differences” in Music: Composition, interpretation and effects. ed. Ivanova T. A. (New York, USA: Nova Science Pub Inc.), 31–56.

[ref23] Dunifon C. M. Rivera S. Robinson C. W. (2016). Auditory stimuli automatically grab attention: evidence from eye tracking and attentional manipulations. J. Exp. Psychol. Hum. Percept. Perform. 42, 1947–1958. 10.1037/xhp0000276, PMID: 27505224

[ref74] Eerola T. Vuoskoski J. K. (2011). A comparison of the discrete and dimensional models of emotion in music. Psychol. Music 39, 18–49. 10.1177/0305735610362821

[ref24] Eriksen C. W. (1995). The flankers task and response competition: a useful tool for investigating a variety of cognitive problems. Vis. Cogn. 2, 101–118. 10.1080/13506289508401726

[ref25] Eriksen B. A. Eriksen C. W. (1974). Effects of noise letters upon the identification of a target letter in a nonsearch task. Percept. Psychophys. 16, 143–149.

[ref26] Fernandez N. B. Hars M. Trombetti A. Vuilleumier P. (2019). Age-related changes in attentional control and their relationship with gait performance in older adults with high risk of falls. Neuroimage 189, 551–559. 10.1016/j.neuroimage.2019.01.030, PMID: 30660655

[ref27] Fernandez N. B. Trost W. J. Vuilleumier P. (2020). Brain networks mediating the influence of background music on selective attention. Soc. Cogn. Affect. Neurosci. 14, 1441–1452. 10.1093/scan/nsaa004, PMID: 31993668 PMC7137722

[ref28] Folstein M. F. Folstein S. E. McHugh P. R. (1975). “Mini-mental state”: a practical method for grading the cognitive state of patients for the clinician. J. Psychiatr. Res. 12, 189–198. 10.1016/0022-3956(75)90026-6, PMID: 1202204

[ref29] Gazzaley A. Cooney J. W. Rissman J. D’esposito M. (2005). Top-down suppression deficit underlies working memory impairment in normal aging. Nat. Neurosci. 8, 1298–1300. 10.1038/nn1543, PMID: 16158065

[ref30] Guerreiro M. J. Murphy D. R. Van Gerven P. W. (2010). The role of sensory modality in age-related distraction: a critical review and a renewed view. Psychol. Bull. 136, 975–1022. 10.1037/a0020731, PMID: 21038938

[ref31] Hasher L. Stoltzfus E. R. Zacks R. T. Rypma B. (1991). Age and inhibition. J. Exp. Psychol. Learn. Mem. Cogn. 17, 163–169. 10.1037//0278-7393.17.1.163, PMID: 1826730

[ref32] Hasher L. Zacks R. T. (1988). “Working memory, comprehension, and aging: a review and a new view” in Psychology of learning and motivation. Vol. 22 ed. Bower G. H. (New York, USA: Elsevier), 193–225.

[ref33] Hsieh S. Fang W. (2012). Elderly adults through compensatory responses can be just as capable as young adults in inhibiting the flanker influence. Biol. Psychol. 90, 113–126. 10.1016/j.biopsycho.2012.03.006, PMID: 22445781

[ref34] Hsieh S. Lin Y. -C. (2014). The boundary condition for observing compensatory responses by the elderly in a flanker-task paradigm. Biol. Psychol. 103, 69–82. 10.1016/j.biopsycho.2014.08.008, PMID: 25168289

[ref35] Hudon C. Potvin O. Turcotte M. -C. D’Anjou C. Dubé M. Préville M. (2009). Normalisation du Mini-Mental State Examination (MMSE) chez les Québécois francophones âgés de 65 ans et plus et résidant dans la communauté. Can. J. Aging 28, 347–357. 10.1017/S0714980809990171 19925700

[ref36] Hunter P. G. Schellenberg E. G. (2010). “Music and emotion” in Music perception. eds. Jones M. R. Fay R. R. Popper A. N. (New York, NY: Springer), 129–164.

[ref75] Husain G. Thompson W. F. Schellenberg E. G. (2002). Effects of musical tempo and mode on arousal, mood, and spatial abilities. Music. Percept. 20, 151–171. 10.1525/mp.2002.20.2.151

[ref37] Jäncke L. Sandmann P. (2010). Music listening while you learn: no influence of background music on verbal learning. Behav. Brain Funct. 6, 1–14. 10.1186/1744-9081-6-3, PMID: 20180945 PMC2828975

[ref38] Jefferies L. N. Smilek D. Eich E. Enns J. T. (2008). Emotional valence and arousal interact in attentional control. Psychol. Sci. 19, 290–295. 10.1111/j.1467-9280.2008.02082.x, PMID: 18315803

[ref39] Jiang J. Scolaro A. J. Bailey K. Chen A. (2011). The effect of music-induced mood on attentional networks. Int. J. Psychol. 46, 214–222. 10.1080/00207594.2010.541255, PMID: 22044234

[ref40] Juslin P. N. Laukka P. (2004). Expression, perception, and induction of musical emotions: a review and a questionnaire study of everyday listening. J. New Music Res. 33, 217–238. 10.1080/0929821042000317813

[ref76] Kallinen K. (2002). Reading news from a pocket computer in a distracting environment: effects of the tempo of background music. Comput. Hum. Behav. 18, 537–551. 10.1016/S0747-5632(02)00005-5

[ref41] Kämpfe J. Sedlmeier P. Renkewitz F. (2010). The impact of background music on adult listeners: a meta-analysis. Psychol. Music 39, 424–448. 10.1177/0305735610376261

[ref77] Kleiner M. Brainard D. Pelli D. (2007). What’s new in Psychtoolbox-3. Perception 36, 1–16.

[ref42] Li K. Z. Lindenberger U. (2002). Relations between aging sensory/sensorimotor and cognitive functions. Neurosci. Biobehav. Rev. 26, 777–783. 10.1016/s0149-7634(02)00073-8, PMID: 12470689

[ref43] Lin F. R. Yaffe K. Xia J. Xue Q. L. Harris T. B. Purchase-Helzner E.. (2013). Hearing loss and cognitive decline in older adults. JAMA Intern. Med. 173, 293–299. 10.1001/jamainternmed.2013.1868, PMID: 23337978 PMC3869227

[ref44] Mammarella N. Fairfield B. Cornoldi C. (2007). Does music enhance cognitive performance in healthy older adults? The Vivaldi effect. Aging Clin. Exp. Res. 19, 394–399. 10.1007/BF03324720, PMID: 18007118

[ref45] Maylor E. A. Lavie N. (1998). The influence of perceptual load on age differences in selective attention. Psychol. Aging 13, 563–573. 10.1037//0882-7974.13.4.563, PMID: 9883457

[ref46] Merritt P. Hirshman E. Wharton W. Stangl B. Devlin J. Lenz A. (2007). Evidence for gender differences in visual selective attention. Pers. Individ. Differ. 43, 597–609. 10.1016/j.paid.2007.01.016

[ref48] Nater U. M. Abbruzzese E. Krebs M. Ehlert U. (2006). Sex differences in emotional and psychophysiological responses to musical stimuli. Int. J. Psychophysiol. 62, 300–308. 10.1016/j.ijpsycho.2006.05.011, PMID: 16828911

[ref49] Nemati S. Akrami H. Salehi S. Esteky H. Moghimi S. (2019). Lost in music: neural signature of pleasure and its role in modulating attentional resources. Brain Res. 1711, 7–15. 10.1016/j.brainres.2019.01.011, PMID: 30629944

[ref78] Nittono H. Tsuda A. Akai S. Nakajima Y. (2000). Tempo of background sound and performance speed. Percept. Mot. Skills 90:1122. 10.2466/PMS.90.3.1122-1122, PMID: 10939056

[ref50] Nosek B. A. Banaji M. R. (2001). The go/no-go association task. Soc. Cogn. 19, 625–666. 10.1521/soco.19.6.625.20886

[ref51] Olsen K. N. Dean R. T. Stevens C. J. Bailes F. (2015). Both acoustic intensity and loudness contribute to time-series models of perceived affect in response to music. Psychomusicology 25, 124–137. 10.1037/pmu0000087

[ref52] Pachana N. A. Byrne G. J. Siddle H. Koloski N. Harley E. Arnold E. (2007). Development and validation of the Geriatric Anxiety Inventory. Int. Psychogeriatr. 19, 103–114. 10.1017/S1041610206003504, PMID: 16805925

[ref54] Rowe G. Hirsh J. B. Anderson A. K. (2007). Positive affect increases the breadth of attentional selection. Proc. Natl. Acad. Sci. U. S. A. 104, 383–388. 10.1073/pnas.0605198104, PMID: 17182749 PMC1765470

[ref55] Russell J. A. (1980). A circumplex model of affect. J. Pers. Soc. Psychol. 39, 1161–1178. 10.1037/h0077714

[ref56] Salimpoor V. N. Benovoy M. Longo G. Cooperstock J. R. Zatorre R. J. (2009). The rewarding aspects of music listening are related to degree of emotional arousal. PLoS One 4:e7487. 10.1371/journal.pone.0007487, PMID: 19834599 PMC2759002

[ref57] Salthouse T. A. (2010). Is flanker-based inhibition related to age? Identifying specific influences of individual differences on neurocognitive variables. Brain Cogn. 73, 51–61. 10.1016/j.bandc.2010.02.003, PMID: 20303636 PMC2862805

[ref58] Savage M. (2006). The musical field. Cult. Trends 15, 159–174. 10.1080/09548960600712975

[ref59] Schubert E. (2004). Modeling perceived emotion with continuous musical features. Music Percept. 21, 561–585. 10.1525/mp.2004.21.4.561

[ref60] Shih Y. -N. Chien W. -H. Chiang H. -S. (2016). Elucidating the relationship between work attention performance and emotions arising from listening to music. Work 55, 489–494. 10.3233/WOR-162408, PMID: 27689591

[ref61] Stoet G. (2010). Sex differences in the processing of flankers. Q. J. Exp. Psychol. 63, 633–638. 10.1080/17470210903464253, PMID: 20013515

[ref62] Theeuwes J. (2010). Top–down and bottom–up control of visual selection. Acta Psychol. 135, 77–99. 10.1016/j.actpsy.2010.02.006, PMID: 20507828

[ref63] Thompson R. G. Moulin C. Hayre S. Jones R. (2005). Music enhances category fluency in healthy older adults and Alzheimer’s disease patients. Exp. Aging Res. 31, 91–99. 10.1080/03610730590882819, PMID: 15842075

[ref64] Thompson W. F. Schellenberg E. G. Husain G. (2001). Arousal, mood, and the Mozart effect. Psychol. Sci. 12, 248–251. 10.1111/1467-9280.00345, PMID: 11437309

[ref65] Trost W. Frühholz S. Schön D. Labbé C. Pichon S. Grandjean D.. (2014). Getting the beat: entrainment of brain activity by musical rhythm and pleasantness. Neuroimage 103, 55–64. 10.1016/j.neuroimage.2014.09.009, PMID: 25224999

[ref66] van den Bosch I. Salimpoor V. Zatorre R. J. (2013). Familiarity mediates the relationship between emotional arousal and pleasure during music listening. Front. Hum. Neurosci. 7:534. 10.3389/fnhum.2013.00534, PMID: 24046738 PMC3763198

[ref67] Vieillard S. Peretz I. Gosselin N. Khalfa S. Gagnon L. Bouchard B. (2008). Happy, sad, scary and peaceful musical excerpts for research on emotions. Cogn. Emot. 22, 720–752. 10.1080/02699930701503567

[ref68] Wahl H. -W. Heyl V. (2003). Connections between vision, hearing, and cognitive function in old age. Generations 27, 39–45.

[ref69] Wahn B. König P. (2017). Is attentional resource allocation across sensory modalities task-dependent? Adv. Cogn. Psychol. 13, 83–96. 10.5709/acp-0209-2, PMID: 28450975 PMC5405449

[ref70] Wild-Wall N. Falkenstein M. Hohnsbein J. (2008). Flanker interference in young and older participants as reflected in event-related potentials. Brain Res. 1211, 72–84. 10.1016/j.brainres.2008.03.025, PMID: 18433737

[ref71] Zeef E. J. Sonke C. J. Kok A. Buiten M. M. Kenemans J. (1996). Perceptual factors affecting age-related differences in focused attention: performance and psychophysiological analyses. Psychophysiology 33, 555–565. 10.1111/j.1469-8986.1996.tb02432.x003, PMID: 8854743

